# BAP31 Facilitates TGF-β Trafficking and Secretion and Promotes Dendritic Cell Dysfunction in Cervical Cancer

**DOI:** 10.3390/cells15191765

**Published:** 2026-09-27

**Authors:** Lin Guo, Yuanli He, Feiming Hu, Yuanjie Sun, Yajie Zhang, Junyi Ren, Ren Zheng, Junqi Zhang, Yubo Sun, Sirui Cai, Yueyue Wang, Kun Yang, Chenchen Hu, Shuya Yang

**Affiliations:** 1 Department of Immunology, Air Force Medical University, No. 169 Changle West Road, Xi’an 710032, China; 2 Yan’an Key Laboratory of Microbial Drug Innovation and Transformation, School of Basic Medicine, Yan’an University, Yan’an 716000, China; 3 School of Medicine, Northwest University, Xi’an 710069, China; 4 School of Basic Medicine, Air Force Medical University, No. 169 Changle West Road, Xi’an 710032, China; 5 Military Medical Innovation Center, Air Force Medical University, Xi’an 710032, China

**Keywords:** cervical cancer, BAP31, TGF-β, dendritic cells, immune microenvironment

## Abstract

Cervical cancer remains one of the major gynecological malignancies worldwide, with tumor immune evasion playing a critical role in disease progression. Although accumulating evidence has implicated BAP31 in tumor progression and immune regulation, its role in immune evasion in cervical cancer remains poorly understood. In this study, we found that BAP31 was significantly upregulated in cervical cancer tissues and that elevated BAP31 expression was associated with poor patient prognosis. Functional assays demonstrated that BAP31 silencing restored the immune activity of dendritic cells. Mechanistically, BAP31 was found to regulate TGF-β at the post-transcriptional level and to facilitate its transport from the endoplasmic reticulum to the Golgi apparatus through an interaction involving the Asn-381 residue, thereby affecting its intracellular maturation and extracellular secretion. Additional functional analyses showed that exogenous TGF-β impaired dendritic cell maturation and migration while enhancing phagocytic activity. Collectively, these findings indicate that BAP31 regulation of TGF-β contributes to dendritic cell dysfunction and the establishment of an immunosuppressive microenvironment in cervical cancer.

## 1. Introduction

Cervical cancer (CC) is the fourth most frequently diagnosed cancer in women worldwide after breast, lung and colorectal cancer [1]. According to GLOBOCAN 2022 estimates, approximately 662,000 new cases and 349,000 deaths from CC occurred globally, with an age-standardized mortality rate of 7.1 per 100,000 people [2]. Although surgery, radiotherapy and chemotherapy remain the main therapeutic strategies for CC, the prognosis of patients with advanced or recurrent disease remains poor [3,4]. Increasing evidence indicates that the tumor immune microenvironment is critical to tumor initiation and progression [5]. Nevertheless, the exact mechanisms by which the immune microenvironment of CC drives malignant progression and immune escape still require further investigation.

B cell receptor-associated protein 31 (BAP31), encoded by the BCAP31 gene, is an evolutionarily conserved and ubiquitously expressed integral endoplasmic reticulum (ER) transmembrane protein with three transmembrane domains and a cytoplasmic C-terminal domain [6,7]. Localized on the ER membrane, BAP31 mediates protein transport from the ER to the Golgi apparatus and participates in essential cellular processes including cell apoptosis and ER-associated degradation [8,9,10]. Recent studies indicate that BAP31 is aberrantly overexpressed in multiple malignancies, promoting tumor cell proliferation, invasion, and migration [11,12]. Notably, BAP31 knockdown has been reported to attenuate transforming growth factor-β (TGF-β)-induced migration and proliferation in lung cancer cells, suggesting a potential functional link between BAP31 and TGF-β related tumor progression [13]. Beyond its roles in tumor progression, BAP31 also modulates immune cell function by influencing BCR and TCR signaling, macrophage polarization, and antigen presentation and is positively correlated with dendritic cell (DC) abundance across cancers [14,15,16]. However, the mechanisms underlying BAP31-mediated regulation of DCs in the tumor immune microenvironment remain unclear.

DCs are professional antigen-presenting cells that initiate antitumor immune responses by activating T lymphocytes [17]. In the tumor microenvironment, soluble factors recruit immature DCs and impede their maturation, among which TGF-β exerts a certain regulatory effect [18]. TGF-β regulates both innate and adaptive immune cells, maintains immune homeostasis, and modulates immune cell development from hematopoietic stem cells [19,20]. Aberrant activation of TGF-β signaling in tumors suppresses antitumor immunity, disrupts immune balance, and promotes immune escape [21,22]. In particular, high TGF-β expression impairs DC maturation, reduces the expression of surface markers such as CD80 and CD86, and compromises the homing ability and therapeutic efficacy of DCs [23]. These findings suggest that TGF-β-mediated DC dysfunction may represent an important mechanism underlying immune escape in CC.

In this study, we investigated the role of BAP31 in regulating DC function in CC and explored the underlying mechanism involving TGF-β trafficking and secretion.

## 2. Materials and Methods

### 2.1. Cell Culture

Human CC cell lines HeLa (Jinyuanbio, Shanghai, China) and SiHa (Jinyuanbio, Shanghai, China) were cultured in DMEM (Corning, NY, USA) with 10% FBS (Biological Industries, Beit Haemek, Israel) and 1% penicillin–streptomycin–amphotericin B (Coolaber, Beijing, China). Human monocytic THP-1 cells (zqxzbio, Shanghai, China) were grown in RPMI 1640 (Corning, NY, USA) supplemented with 10% FBS (zqxzbio, Shanghai, China), 1% antibiotics and 0.05 mM β-mercaptoethanol (Aladdin, Shanghai, China). All cells were cultured at 37 °C in a humidified atmosphere with 5% CO_2_.

### 2.2. Sample Collection

A human CC tissue microarray (TMA) was purchased from Xi’an Taibosi Biotechnology Co., Ltd. (Xi’an, China), containing tumor tissue specimens from 100 patients with CC. The diagnosis of CC was based on histopathological examination. The corresponding clinicopathological characteristics and follow-up information were provided by the commercial supplier. Each tissue core was derived from an independent patient. Clinical information, including age, histological grade, tumor stage, and other clinicopathological parameters, was obtained from available medical records. All patient-identifiable information and tissue-associated data were de-identified. This retrospective study using a commercially obtained TMA was performed for scientific research purposes only. A total of 14 specimens were excluded following staining and quality assessment, including 5 cases due to tissue detachment and 9 due to tissue loss during multiple rounds of immunostaining, resulting in incomplete evaluation of some markers. Finally, 86 specimens with satisfactory staining quality and complete evaluable data were included in the subsequent analyses. Multiplex immunofluorescence (mIF) staining was performed to simultaneously detect BAP31, TGF-β, and CD11c expression in the included tissue specimens. Complete images of each tissue core were acquired, and the entire evaluable tissue area was used for quantitative analysis. The expression levels of BAP31, TGF-β, and CD11c were quantified based on the percentage of positive cells and used for subsequent statistical analyses. For clinicopathological analysis, the median percentage of BAP31-positive cells within the tissue microarray cohort was used as the cutoff value to stratify the 86 patients into BAP31-high and BAP31-low groups, with 43 patients in each group. The clinicopathological characteristics of patients stratified according to BAP31 expression levels are summarized in Table 1. This study was conducted in accordance with the ethical guidelines for research involving human participants and approved by the Ethics Committee of Liaocheng Second People’s Hospital (Approval No. KB2025-Ethics-48-01) and the Institutional Review Board of the First Affiliated Hospital of Air Force Medical University (Approval No. KY20253725-1).

### 2.3. Bioinformatics Analysis

Gene expression and clinical data were obtained from The Cancer Genome Atlas (TCGA) and Genotype-Tissue Expression (GTEx) databases. For BAP31 expression analysis, a total of 304 CC tissue samples and 13 normal cervical tissue samples were included. Survival analysis of BAP31 was performed using available BAP31 expression and clinical follow-up data from the TCGA-CESC cohort in the R environment with the survminer (version 0.4.9) and survival (version 3.5.7) packages. Kaplan–Meier survival curves were generated, differences between groups were assessed using the log-rank test, and hazard ratios (HRs) with 95% confidence intervals (CIs) were calculated. A *p* value < 0.05 was considered statistically significant. The GSE9750 and GSE29570 datasets from the Gene Expression Omnibus (GEO) database were used to validate BAP31 expression. TCGA-CESC samples were divided into BAP31-high and BAP31-low groups according to the median BAP31 expression level. Differentially expressed genes (DEGs) were identified using the DESeq2 R package (version 1.48.1), with |log2FC| > 0.5 and FDR < 0.05 as the cutoff criteria. Kyoto Encyclopedia of Genes and Genomes (KEGG) pathway enrichment analysis was performed using the clusterProfiler R package, with a *p* value < 0.05 considered statistically significant. The prognostic significance of TGF-β1 expression was evaluated using the Kaplan–Meier Plotter. A total of 304 patients with cervical squamous cell carcinoma were stratified into TGF-β1-high and TGF-β1-low groups based on percentile ranking. Overall survival was compared using the log-rank test, and HRs with 95% CIs were calculated. The GSCA and TISIDB databases and the xCell algorithm were used to assess immune cell infiltration in CC tissues. For xCell analysis, gene expression data from the TCGA-CESC cohort were used to estimate the relative enrichment scores of immune and stromal cell populations. Samples were divided into TGF-β1-high and TGF-β1-low groups according to the median TGF-β1 expression level. Differences in xCell enrichment scores between the two groups were assessed using the Wilcoxon rank-sum test. Spearman’s rank correlation analysis was used to evaluate the association between gene expression and immune cell infiltration levels.

### 2.4. RNA Interference

Small interfering RNAs (siRNAs) targeting BAP31 were purchased from Tsingke Biological Technology (Beijing, China). siRNA transfection was carried out using Lipofectamine 3000 (Invitrogen, Carlsbad, CA, USA). The transfection efficiency was confirmed by Quantitative Real-Time Reverse Transcription PCR (qRT-PCR) and Western blot (WB) analysis. All siRNA sequences are presented in Table S1.

### 2.5. Generation of Conditioned Media

To prepare tumor cell-conditioned medium (CM), 2 × 10^5^ tumor cells were seeded into 6-well plates under identical culture conditions. Cells were assigned to the negative control group (NC) and BAP31-knockdown group, in which cells were transfected with small interfering RNAs targeting BAP31 (siBAP31). The NC was transfected with negative control siRNA, and the siBAP31 group was transfected with BAP31-targeted siRNA by lipofection. Six hours after transfection, the medium was changed to fresh complete medium. After the cells reached stable growth, they were cultured for another 48 h. After collection, the supernatants were filtered with 0.22 μm sterile PES syringe filters (Bioland, Hangzhou, China) to acquire tumor cell-conditioned media of distinct treatment groups.

### 2.6. qRT-PCR

Total RNA was extracted from samples of each treatment group with an RNA extraction kit. One-step reverse transcription was then conducted to synthesize complementary DNA (cDNA). qRT-PCR was carried out using SYBR Green qPCR Master Mix (Vazyme Biotech, Nanjing, China). All primer sequences used in this study are shown in Supplementary Table S2.

### 2.7. WB and Immunoprecipitation (IP) Assays

Protein expression levels of BAP31 and TGF-β were analyzed by WB. CC cells were lysed in RIPA buffer containing protease inhibitors (Beyotime Biotechnology, Shanghai, China) for 10 min. After centrifugation at 12,000× *g* for 10 min at 4 °C, the supernatants were collected, and protein concentrations were determined using a BCA assay kit (Thermo Fisher Scientific, Waltham, MA, USA). Proteins were separated by SDS-PAGE, transferred onto membranes at 0.4 A for 30 min, blocked with 5% non-fat milk, and incubated with primary antibodies overnight at 4 °C. After incubation with secondary antibodies, protein bands were visualized using Chemistar™ High-sig ECL substrate (Tanon, Shanghai, China). Detailed information on the antibodies used in this experiment is shown in Supplementary Table S3.

For the Co-IP assay, cell lysis was performed using a lysis buffer containing protease and phosphatase inhibitors. After centrifugation and the collection of supernatants, laboratory-prepared mouse monoclonal antibody against BAP31 (FMU-BAP31-2), an IgG control antibody and Protein A/G agarose beads (Santa Cruz Biotechnology, Dallas, TX, USA) were added and incubated overnight. After washing the agarose beads, interacting proteins were detected by WB.

The His-tagged full-length and truncated BAP31 plasmids were constructed by GeneCreate (Wuhan, China). The Flag-tagged TGF-β mutant plasmids with mutations at key amino acid sites (ASN-381, CYS-33) were constructed and sequence-verified by Tsingke Biotechnology. These plasmids were transfected into HeLa cells using Lipofectamine 3000. Seventy-two hours after transfection, His-tag and Flag-tag immunomagnetic beads were used separately to enrich interacting proteins by overnight incubation at 4 °C. The eluted proteins were further analyzed via WB.

### 2.8. IF

Cells were seeded into 15 mm glass-bottomed culture dishes and cultured until reaching 30% confluence. Cells were washed three times with PBS before fixation with 4% paraformaldehyde and then permeabilized with 0.2% Triton X-100 for 10 min. Staining was performed using the Vari Fluor 4-color multiplex IF kit (#HY-KD1004, MCE, New York, NY, USA). Briefly, cells were incubated with 3% H_2_O_2_ for 15 min at room temperature to block endogenous peroxidase activity, washed three times with PBS, and blocked with 3% BSA for 30 min at 37 °C. After overnight incubation with primary antibodies at 4 °C, cells were washed three times with PBS and incubated with HRP-conjugated secondary antibodies from the kit for 50 min at room temperature in the dark. TSA signal amplification was then performed followed by three PBS washes. For multiplex staining, previous antibodies were stripped using antibody stripping buffer (#G1266-30ML, Servicebio, Wuhan, China) at 37 °C for 15 min, and the staining procedure was repeated. Nuclei were counterstained with DAPI, and all images were acquired and analyzed using an FV3000 confocal microscope (Olympus, Tokyo, Japan). Details of the antibodies used are listed in Table S4.

### 2.9. Enzyme-Linked Immunosorbent Assay (ELISA)

TGF-β levels in cell culture supernatants were determined using a human TGF-β1 ELISA kit (#abs552208, Absin, Shanghai, China) according to the manufacturer’s instructions. Before measurement, culture supernatants were acidified with 1 N HCl at room temperature for 10 min and subsequently neutralized with 1 N NaOH. Following acid activation and neutralization, total TGF-β1 levels were quantified by ELISA.

### 2.10. Molecular Docking Analysis

Protein–protein interaction modeling between BAP31 and TGF-β1 was performed using an AlphaFold-based multimer modeling workflow. The amino acid sequences of human BAP31 (CAA57415.1) and TGF-β1 precursor (NP_000651.3) were obtained from the National Center for Biotechnology Information (NCBI) database and used as inputs for complex structure prediction. The predicted models were evaluated based on the predicted interface TM-score (ipTM), predicted TM-score (pTM), and ranking score. The selected model showed an ipTM of 0.28, a pTM of 0.36, and a ranking score of 0.45. The predicted complex structure was visualized using PyMOL software (version 3.0).

### 2.11. Inducing Differentiation of Mature DCs

A THP-1-derived DC model was used as a reproducible in vitro model for subsequent functional experiments, based on a previously established differentiation protocol [24]. THP-1 cells were collected by centrifugation, adjusted to a density of 2 × 10^5^ cells/mL with complete RPMI 1640 medium, and then seeded into six-well plates. Cells were induced to differentiate with 100 ng/mL IL-4 (#HY-P70445, MCE, New York, NY, USA) and 100 ng/mL GM-CSF (#HY-P7016A, MCE, New York, NY, USA). Fresh medium supplemented with identical cytokines was replaced every two days. After continuous culture for 5 days, the cells differentiated into immature dendritic cells (iDCs). To generate a mature DC (mDC) positive-control population, a subset of iDCs were resuspended in serum-free medium at a density of 2 × 10^5^ cells/mL and supplemented with 200 ng/mL IL-4, 100 ng/mL GM-CSF, 20 ng/mL TNF-α (#HY-P70426A, MCE, New York, NY, USA), and 200 ng/mL ionomycin (#HY-13434, MCE, New York, NY, USA). The cells were then cultured for an additional 48 h at 37 °C in a humidified atmosphere containing 5% CO_2_. TNF-α and ionomycin were used as maturation stimuli to promote the acquisition of an mDC phenotype. A parallel population of iDCs maintained under the same culture conditions but without TNF-α and ionomycin served as the iDC control. DC maturation was subsequently assessed by flow cytometric analysis of the maturation-associated surface markers CD83, CD86, and HLA-DR. During the differentiation process, morphological changes were monitored using an EVOS M5000 imaging system (Thermo Fisher Scientific, Waltham, MA, USA) equipped with a 20× objective under identical imaging settings across all experimental groups. Representative images were acquired to evaluate characteristic morphological changes associated with DC-like differentiation, including increased cell size, irregular cell morphology, and the formation of dendrite-like extensions.

### 2.12. Co-Culture of DCs with Tumor CM

After iDCs were generated, the original culture medium was removed. Tumor CM from the indicated treatment groups was mixed with complete RPMI 1640 medium at a 1:1 volume ratio and applied to iDCs. For the rescue experiments, recombinant human TGF-β (#HY-P70543, MCE, New York, NY, USA) was added directly to the DC culture immediately after application of the corresponding CM, at a final concentration of 0.2 ng/mL. The experimental groups included (1) NC CM, (2) siBAP31 CM, (3) siBAP31 CM supplemented with recombinant TGF-β, and (4) recombinant TGF-β alone. Cells were incubated at 37 °C with 5% CO_2_ for 48 h and subsequently collected for phenotypic and functional analyses.

### 2.13. Flow Cytometry

A total of 5 × 10^5^ cells were collected, washed twice with ice-cold PBS containing 5% fetal bovine serum, and subsequently incubated with fluorochrome-conjugated antibodies for 30 min at 4 °C in the dark. Samples were acquired using an Agilent ACEA NovoCyte 3000RYB flow cytometer (Agilent Technologies, Santa Clara, CA, USA) and analyzed using NovoExpress software (v1.4.1). For flow cytometric analysis, cells were first gated based on forward scatter (FSC) and side scatter (SSC) to exclude debris, followed by FSC-A versus FSC-H gating to remove doublets. Unstained controls were used to determine baseline fluorescence, while matched isotype controls were used to assess nonspecific staining and assist with gating. The same gating strategy was applied consistently to all samples. For quantitative analysis of DC maturation markers, relative mean fluorescence intensity (MFI) was calculated by dividing the MFI of each stained sample by the MFI of the unstained control, which was used as the background fluorescence reference. For phenotypic analysis, the antibodies used included PE-conjugated anti-CD83, APC-conjugated anti-HLA-DR, PE/Cyanine7-conjugated anti-CD86, and FITC-conjugated anti-CD11b antibodies (Elabscience, Wuhan, China), as well as PE-conjugated anti-CD141 antibody (Selleck Chemicals, Houston, TX, USA). Detailed information on the antibodies and their corresponding isotype controls is provided in Supplementary Table S5.

### 2.14. Phagocytosis Assay

Cells were collected and resuspended in RPMI 1640 medium containing 10% fetal bovine serum. FITC-dextran (#HY-128868D, MCE, New York, NY, USA) was added to a final concentration of 1 mg/mL, and the cells were incubated at 37 °C and 4 °C. After 120 min of incubation, the cells were washed twice with ice-cold PBS and detected using the NovoCyte Advanteon flow cytometer. The results are presented as ΔMFI, which was calculated by subtracting the background MFI of cells incubated at 4 °C from the MFI of cells incubated at 37 °C.

### 2.15. Cell Migration Assays

Transwell migration assays were performed using 24-well Transwell inserts (Thermo Fisher Scientific, Waltham, MA, USA). A total of 2 × 10^5^ DCs were resuspended in 200 μL of serum-free medium and seeded into the upper chamber, while tumor CM from the indicated treatment groups was added to the lower chamber as the chemoattractant. After incubation at 37 °C for 24 h, non-migrated cells remaining on the upper surface of the membrane were gently removed with a cotton swab. Cells that had migrated to the lower surface of the membrane were fixed with 4% paraformaldehyde and stained with 0.1% crystal violet. Images were acquired using an EVOS M5000 imaging system with a 20× objective under identical imaging settings across all experimental groups. Five randomly selected, non-overlapping fields were imaged for each insert, and migrated cells were quantified in a blinded manner using ImageJ software (v1.47t). Three replicate wells were included for each experimental group, and the experiment was independently repeated at least three times.

### 2.16. Statistical Analysis

Statistical analyses were performed using GraphPad Prism version 10.6.0. For comparisons between two groups, Student’s *t*-test was used. For comparisons among three or more groups, one-way analysis of variance (ANOVA) followed by Tukey’s multiple comparisons test was performed. Each experiment was independently repeated at least three times, and quantitative data are presented as the mean ± standard deviation (SD). Statistical significance is indicated as follows: * *p* < 0.05, ** *p* < 0.01, *** *p* < 0.001, **** *p* < 0.0001; ns, not significant.

## 3. Results

### 3.1. BAP31 Is Overexpressed in CC and Linked to Poor Patient Prognosis

To explore the expression characteristics of BAP31, we first performed pan-cancer expression profiling. The results showed that BAP31 is aberrantly overexpressed across multiple malignancies, implying a potential broad role in regulating tumor progression (Figure 1A). Based on the above results, we further focused on CC to analyze BAP31 expression levels. Validation using TCGA and GTEx datasets confirmed that BAP31 was significantly upregulated in CC tissues (Figure 1B). Survival analysis showed that patients with high BAP31 expression exhibited markedly shorter overall survival (Figure 1C), indicating that elevated BAP31 was closely associated with poor prognosis in CC. We further validated these findings using public databases (GSE9750, GSE29570), which confirmed elevated BAP31 expression in CC tissues (Figure 1D,E). mIF staining of 86 CC tissue samples was performed to assess the association between BAP31 expression and clinicopathological features. For clinicopathological analysis, patients were stratified into BAP31-high and BAP31-low groups according to the median BAP31 expression level within the tissue microarray cohort. The proportion of patients with high BAP31 expression was significantly greater among those with advanced clinical stage and higher T classification (Table 1). Consistently, tissue microarray analysis demonstrated that BAP31 expression progressively increased with tumor stage (Figure 1F,G). Collectively, these findings suggest that BAP31 acts as a crucial oncogenic regulator in CC.

### 3.2. BAP31 Expression Correlates with DC Infiltration in CC and Supports DC Model Establishment

Tumor immune escape is a core mechanism driving CC progression, and immune cells play vital regulatory roles in this process [25]. To investigate the potential relationship between BAP31 and the immune microenvironment in CC, we first analyzed the correlation between BAP31 expression and the infiltration levels of 24 immune cell types in the tumor microenvironment using the GSCA database (Figure 2A). DC infiltration showed a relatively strong positive correlation with BAP31 expression (Figure 2B). Subsequently, we examined this association using the TISIDB database and obtained consistent results (Figure 2C,D). To further evaluate this association in clinical samples, mIF staining was performed on the CC tissue microarray, and all 86 evaluable CC tissue specimens were included in the mIF analysis. Obvious co-localization of BAP31 and DCs was observed (Figure 2E), and BAP31 expression was significantly positively correlated with CD11c^+^ levels, consistent with bioinformatics results (Figure 2F). These results suggest an association between BAP31 expression and DC abundance in CC, indicating a potential role of BAP31 in regulating the tumor immune microenvironment. We next established an in vitro model for subsequent functional experiments. THP-1 monocytes were induced to differentiate into DC-like cells according to an established protocol (Figure 2G). Flow cytometry showed that CD11b and CD141 expression was significantly upregulated in the induced cells, supporting the establishment of the THP-1-derived DC model (Figure 2H). In addition, CD83, CD86, and HLA-DR were used to assess DC maturation, and the expression levels of these maturation-associated markers were markedly higher in mDCs than in iDCs. This model was then used to investigate the regulatory effect of the CC cell CM on DC maturation.

### 3.3. BAP31 Silencing in CC Cells Modulates DC Maturation and Function

To explore the regulatory effect of BAP31 on the biological functions of DCs and to mimic DC regulation within the CC tumor microenvironment, we established an in vitro culture system using tumor CM. The generated iDCs were treated for 48 h with CM collected from NC- or siBAP31-transfected HeLa and SiHa cells. Flow cytometry was subsequently performed to assess DC maturation based on the expression of the maturation-associated surface markers CD83, CD86, and HLA-DR. The results revealed that CM derived from BAP31-knockdown cells markedly upregulated the expression of the above activation markers in DCs compared with the NC (Figure 3A,B). DC migration is essential for tumor recognition, tumor microenvironment infiltration and initiation of local anti-tumor immune responses [26]. Therefore, Transwell assays were performed to characterize the migratory response of DCs to CM derived from NC-or BAP31-silenced CC cells (Figure 3C–E). Compared with NC CM, CM derived from BAP31-silenced HeLa and SiHa cells elicited a significantly greater migratory response, as evidenced by the increased number of migrated DCs. Phagocytic function is a key indicator for assessing the biological activity of DCs. Under physiological conditions, iDCs possess stronger phagocytic ability, which shows a clear decline as cells mature [27]. We detected DC phagocytic activity via the FITC-dextran uptake assay. Flow cytometry showed that DCs in the siBAP31 group exhibited a lower change in mean fluorescence intensity and reduced dextran uptake compared with those in the NC (Figure 3F,G). In conclusion, BAP31 modulates the phenotypic maturation, migration and antigen phagocytic function of DCs in CC tumors.

### 3.4. BAP31 is Associated with TGF-β Expression, Prognosis, and Protein Secretion in CC

BAP31 is capable of regulating the biological behavior of DCs, yet its precise molecular mechanism remains unclear. To further explore the downstream signaling network regulated by BAP31, we retrieved transcriptomic data from the TCGA database. Samples were divided into BAP31-high and -low expression groups according to the median BAP31 expression level, followed by differential gene expression analysis (Figure 4A). The identified differentially expressed genes were subsequently subjected to pathway enrichment analysis (Figure 4B). KEGG enrichment analysis revealed significant enrichment of the TGF-β signaling pathway among the DEGs identified between the BAP31-high and -low groups, suggesting a potential functional association between BAP31 and TGF-β signaling. Therefore, TGF-β was selected as a candidate molecule for subsequent clinical and cellular validation. mIF staining on CC tissue microarrays demonstrated that TGF-β expression increased gradually with the progression of tumor stage (Figure 4C,D). To evaluate the prognostic significance of TGF-β expression, we performed overall survival analysis using the Kaplan–Meier Plotter. Kaplan–Meier curves showed that patients with high TGF-β1 expression had significantly poorer overall survival than those with low TGF-β1 expression (Figure 4E). We carried out cellular experiments to further clarify the regulatory relationship between BAP31 and TGF-β. qRT-PCR showed that TGF-β mRNA levels did not change significantly after BAP31 knockdown (Figure 4F). WB and ELISA analyses (Figure 4G–I) showed accumulation of TGF-β protein within the cells, accompanied by increased intracellular TGF-β levels and decreased TGF-β levels in the culture supernatant. These findings suggest that BAP31 influences TGF-β at the post-transcriptional level, potentially by affecting its intracellular trafficking and secretion rather than its transcription.

### 3.5. BAP31 Regulates TGF-β Trafficking and Maturation Through an Asn-381 Interaction

As a molecular chaperone, BAP31 binds to target proteins and transports them to the Golgi apparatus, thereby participating in the regulation of protein maturation and functional activation [10]. Co-IP assays demonstrated an intracellular association between BAP31 and TGF-β (Figure 5A). Molecular docking analysis further predicted a potential interaction interface between the two proteins involving specific amino acid residues (Figure 5B). To investigate the potential contribution of these residues to the BAP31–TGF-β association, we generated TGF-β point mutants at Asn-381 and Cys-33 and subsequently performed co-IP assays. Compared with wild-type TGF-β, the Asn-381 mutation markedly reduced the co-immunoprecipitation of TGF-β with BAP31, whereas the effect of the Cys-33 mutation was less pronounced. These results suggest that Asn-381 contributes to the association between BAP31 and TGF-β (Figure 5C). IF staining showed obvious co-localization of BAP31 and TGF-β in CC cells, further confirming their stable intracellular interaction (Figure 5D). Given that BAP31 acts as both a molecular chaperone and a protein transporter, we next explored the TGF-β trafficking pathway mediated by BAP31. IF analysis indicated that after BAP31 knockdown, TGF-β failed to be transported to the Golgi apparatus in HeLa cells. Hardly any co-localization signal was detected between TGF-β and the Golgi apparatus, and most TGF-β was retained in the ER. This phenotype was also recapitulated in SiHa cells (Figure 5E, Figure S1A,B). We further compared the subcellular localization of wild-type TGF-β and the ASN-381 mutant in HeLa cells using IF (Figure 5F). Wild-type TGF-β was normally delivered to the Golgi apparatus, whereas the ASN-381 mutant was predominantly trapped in the ER with negligible co-localization with the Golgi apparatus. Additionally, tissue microarray analysis of clinical samples showed prominent co-localization of BAP31 and TGF-β (Figure 5G), and their expression levels were positively correlated (Figure 5H). Collectively, these findings suggest that BAP31 facilitates the trafficking of TGF-β from the endoplasmic reticulum to the Golgi apparatus, thereby supporting its maturation and functional activation.

### 3.6. Functional Contribution of TGF-β to DC Regulation in CC

TGF-β is a major immunosuppressive cytokine driving tumor immune escape [21]. To further characterize the relationship between TGF-β and DCs, we assessed the association between TGF-β expression and DC infiltration as well as its effects on DC function. xCell analysis revealed distinct immune infiltration profiles between CC tissues exhibiting high versus low TGF-β expression, and TGF-β levels were positively correlated with DC infiltration (Figure 6A,B). Consistent findings were obtained from GSCA database analysis (Figure S2A,B). mIF analysis of CC tissue microarrays further demonstrated spatial co-localization of TGF-β and CD11c^+^ cells, and TGF-β expression was positively correlated with CD11c^+^ cell density (Figure 6C,D). We subsequently evaluated the effects of TGF-β on the functional phenotypes of DCs. Compared with the NC CM group, exogenous TGF-β treatment reduced the expression of the DC maturation markers CD83, CD86, and HLA-DR. Supplementation with recombinant TGF-β under the siBAP31 CM condition partially reversed the DC maturation phenotype changes associated with BAP31 knockdown (Figure 6E,F). Transwell migration assays further showed that TGF-β treatment altered the number of migrated DCs (Figure 6G,H). In addition, the phagocytic activity of DCs was evaluated using the FITC-dextran uptake assay. TGF-β treatment increased FITC-dextran uptake in DCs, suggesting that TGF-β modulates their phagocytic function (Figure 6I,J). Collectively, these findings indicate that TGF-β regulates DC functional phenotypes and support a functional contribution of TGF-β to BAP31-associated regulation of DC function.

## 4. Discussion

CC is a highly prevalent and lethal gynecological malignancy worldwide [28]. Patients with advanced and recurrent diseases generally have poor clinical outcomes, and tumor immune escape is the key factor responsible for this therapeutic dilemma and unfavorable prognosis [29,30,31,32].

Studies have shown that BAP31, an endoplasmic reticulum transmembrane protein, is aberrantly overexpressed in a variety of solid malignant tumors. In gastric cancer, BAP31 specifically binds to cyclin-dependent kinase inhibitor p27kip1 and regulates its degradation, thereby accelerating tumor cell proliferation [33]. In hepatocellular carcinoma, BAP31 promotes tumor metastasis by activating the AKT/β-catenin signaling pathway [34]. In colorectal cancer, p20BAP31 induces mitochondrial apoptosis via the ROS/JNK pathway and modulates PERK-mediated endoplasmic reticulum stress and autophagy [35]. Consistent with these pan-cancer findings, our study verified that BAP31 is significantly upregulated in CC tissues. High expression of BAP31 is closely associated with clinical stage and shortened overall survival in patients, suggesting that BAP31 is involved in CC progression and has potential prognostic relevance.

In addition to regulating the intrinsic malignant phenotypes such as tumor cell proliferation and metastasis, BAP31 exerts immunomodulatory effects. Existing studies have confirmed that BAP31 can activate multiple innate immune cells. In microglia, BAP31 modulates reactive oxygen species levels through the p22phox and Keap1/Nrf2/HO-1 pathways to trigger inflammatory responses [36]. Under the pathological condition of sepsis, BAP31 facilitates adhesion between macrophages and neutrophils, induces M1 macrophage polarization, and increases the secretion of pro-inflammatory cytokines including IL-1β and TNF-α via the NF-κB signaling pathway [37]. Nevertheless, the above studies only focused on inflammation-related innate immune cells, and the regulatory role of BAP31 in key antigen-presenting cells during anti-tumor immune responses remains unreported. DCs are the most potent professional antigen-presenting cells in the body and play a central role in initiating anti-tumor immune responses. Their functional impairment is a major cause of tumor immune escape [38,39,40]. To our knowledge, our study provides evidence that BAP31 modulates DC function in CC, which may contribute to an immunosuppressive tumor microenvironment and facilitate immune evasion. These findings suggest a potential role of BAP31 in tumor immune regulation.

TGF-β is a vital multifunctional immunosuppressive cytokine. Its critical roles in tumor microenvironment remodeling, immune homeostasis disruption and malignant tumor progression have been well documented [19]. Accumulating evidence has demonstrated that aberrant activation of the TGF-β signaling pathway acts as a core regulator in the immune escape of various malignancies. In EGFR-mutant non-small-cell lung cancer, elevated TGF-β mediated by the ERK1/2-p90RSK pathway suppresses the infiltration, proliferation and cytotoxic function of CD8+ T cells, forming a cold tumor immune microenvironment [41]. Park et al. demonstrated that TGF-β markedly upregulates PD-1 expression on the surface of tumor-infiltrating T cells via the downstream transcription factor Smad3, thereby inducing T cell exhaustion and mediating tumor immune evasion [42]. Lee et al. demonstrated that TGF-β upregulates matrix metalloproteinase 2 expression, which induces the shedding of NKG2D ligands on lung cancer cells at the post-transcriptional level and further suppresses NK cell-mediated anti-tumor immunity [43]. On this basis, our study demonstrated that TGF-β is highly expressed in CC tissues and that its maturation, secretion, and biological activity are closely associated with BAP31-mediated endoplasmic reticulum-to-Golgi transport. As a molecular chaperone, BAP31 interacts with TGF-β at the Asn-381 residue and participates in its intracellular trafficking and maturation, thereby facilitating the accumulation of functional TGF-β within the tumor microenvironment. Further functional analyses showed that exogenous TGF-β impaired DC maturation and function, suggesting that TGF-β contributes to the maintenance of an immunosuppressive DC phenotype and promotes the establishment of an immunosuppressive microenvironment in CC.

## 5. Conclusions

In conclusion, both BAP31 and TGF-β are aberrantly overexpressed in CC tissues. In vitro functional experiments showed that BAP31 knockdown improves DC function. Mechanistically, BAP31 is associated with TGF-β and facilitates its trafficking from the ER to the Golgi apparatus, as well as its intracellular maturation and extracellular secretion. Moreover, exogenous TGF-β impaired DC maturation and migratory capacity while enhancing phagocytic activity, suggesting that DCs may be maintained in a relatively immature state. Taken together, these results suggest that BAP31-mediated regulation of TGF-β contributes to DC dysfunction and the development of an immunosuppressive tumor microenvironment in CC.

## Figures and Tables

**Figure 1:**
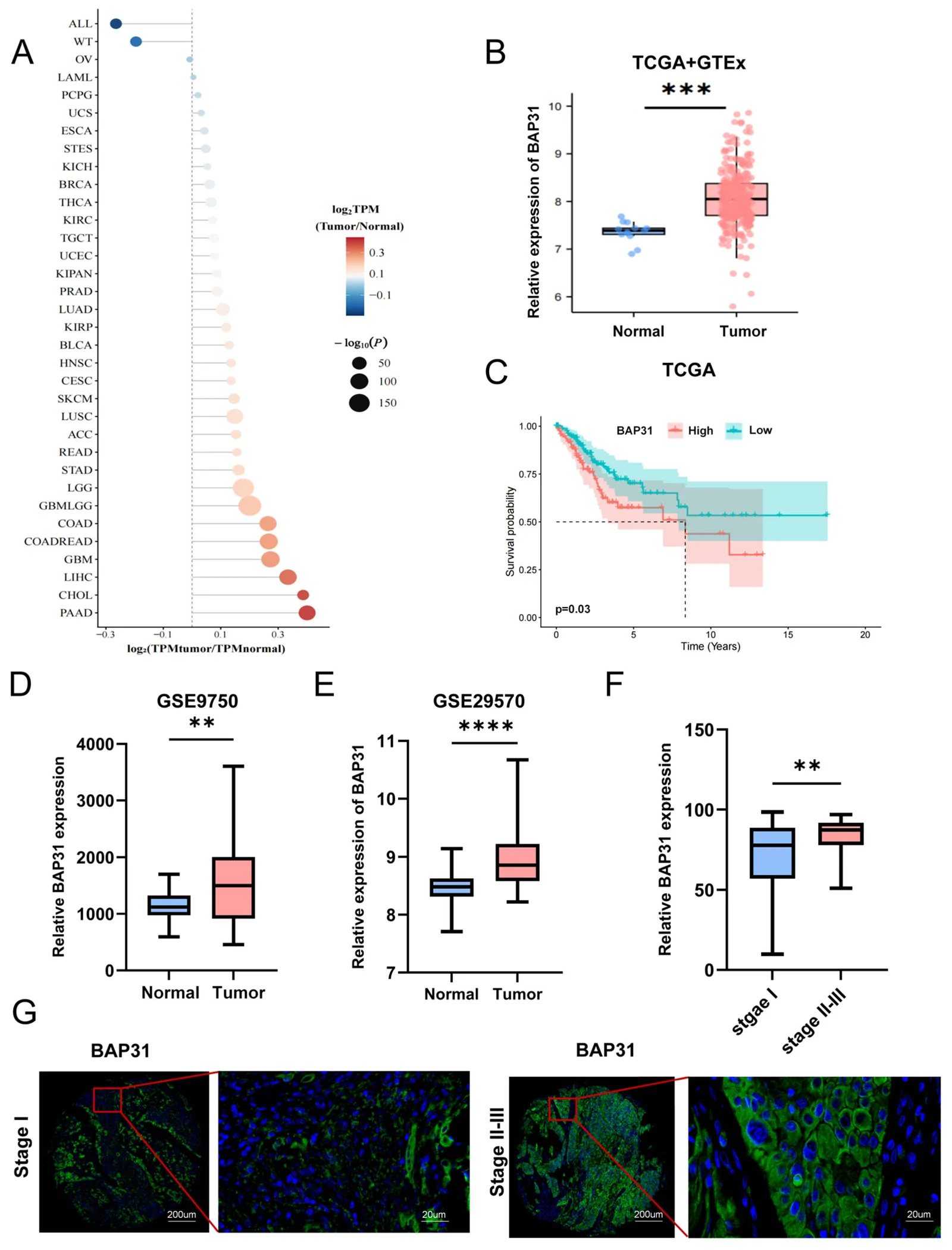
BAP31 is overexpressed in CC and linked to poor patient prognosis. (**A**) Bubble plot showing the differential expression of BAP31 between tumor tissues and paired normal tissues across different cancer types in the TCGA database. (**B**) Comparison of BAP31 expression between normal cervical tissues (*n* = 13) and CC tissues (*n* = 304) from the GTEx and TCGA-CESC databases. (**C**) Kaplan–Meier overall survival analysis of TCGA patients according to BAP31 expression (*n* = 304). Patients were stratified into BAP31-high and BAP31-low groups according to the median BAP31 expression level (HR = 1.35, 95% CI: 1.16–2.45; log-rank *p* = 0.03). The horizontal dashed line indicates a 50% survival probability, and the vertical dashed lines mark the corresponding median survival time points. (**D**) BAP31 expression in normal (*n* = 24) and CC tissues (*n* = 42) from the GSE9750 dataset. (**E**) BAP31 expression in normal (*n* = 17) and CC tissues (*n* = 45) from the GSE29570 dataset. (**F**) Comparison of BAP31 expression between Stage I (*n* = 61) and Stage II–III (*n* = 25) CC tissues from the tissue microarray cohort. (**G**) Representative mIF staining of BAP31 expression in CC tissues at different clinical stages (*n* = 86). ** *p* < 0.01, *** *p* < 0.001, **** *p* < 0.0001.

**Figure 2:**
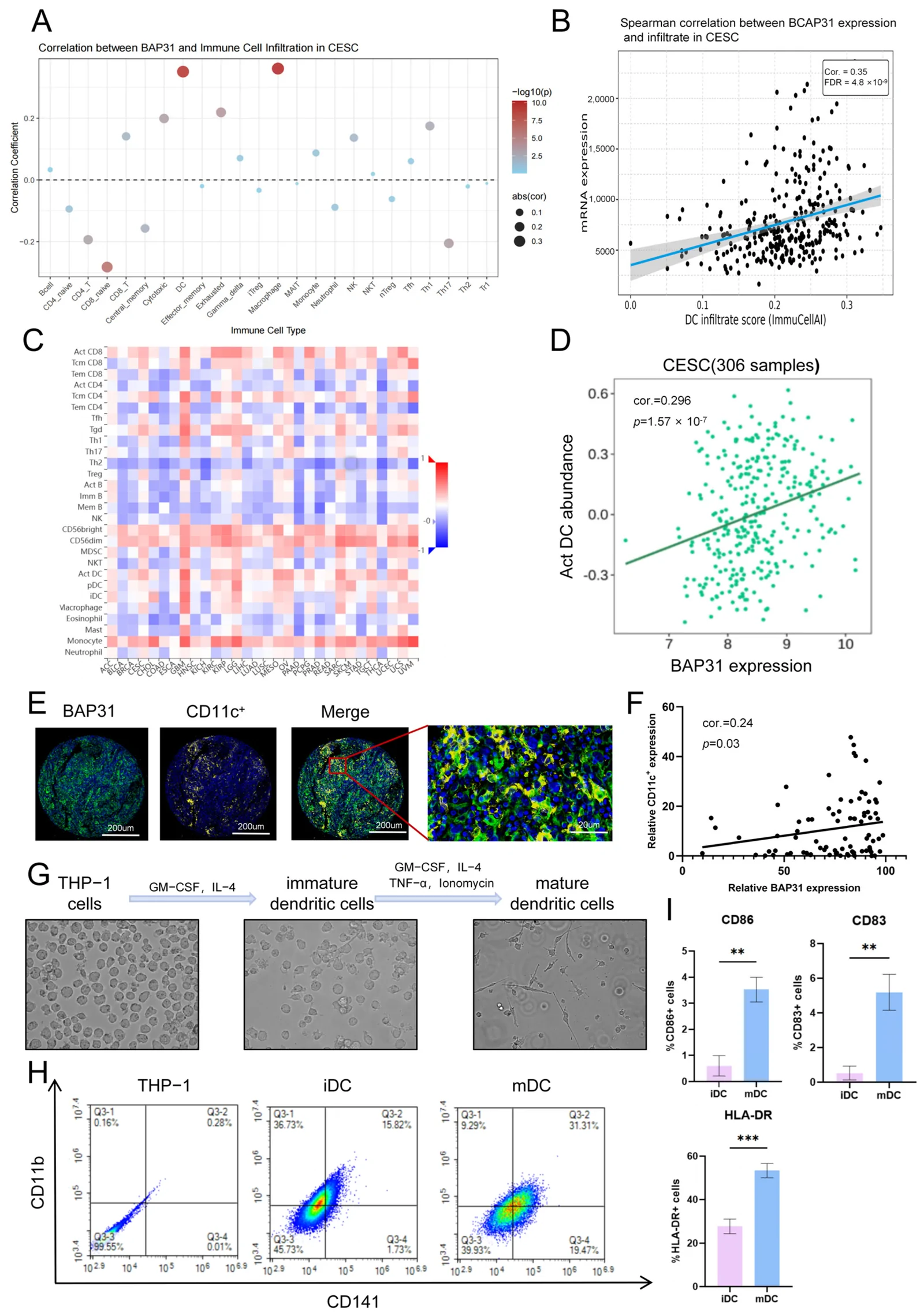
BAP31 expression correlates with DC infiltration in CC and supports DC model establishment. (**A**) Dot plot showing the correlation between BAP31 expression and infiltration levels of 24 immune cell types in CC samples from the GSCA database. (**B**) Correlation between BAP31 mRNA expression and DC infiltration in the GSCA database. Correlation was assessed using Spearman’s rank correlation analysis (*n* = 310). (**C**) Pan-cancer correlation heatmap of BAP31 expression and activated DC infiltration based on the TISIDB database. (**D**) Correlation between BAP31 expression and activated DC infiltration in CC (*n* = 306). Correlation was assessed using Spearman’s rank correlation analysis. (**E**) Representative mIF images of CC tissues. Tissue sections were co-stained for BAP31 (green) and the DC marker CD11c^+^ (yellow), and cell nuclei were counterstained with DAPI (blue) (*n* = 86). (**F**) Correlation between BAP31 expression and CD11c^+^ cell density in CC tissues (*n* = 86). Correlation was assessed using Spearman’s rank correlation analysis. (**G**) Representative morphological changes in THP-1 cells during differentiation into iDCs and mDCs. (**H**) Flow cytometry analysis of DC markers CD11b and CD141 in THP-1 cells, iDCs, and mDCs. (**I**) Quantification of CD83, CD86, and HLA-DR expression in iDCs and mDCs. ** *p* < 0.01, *** *p* < 0.001.

**Figure 3:**
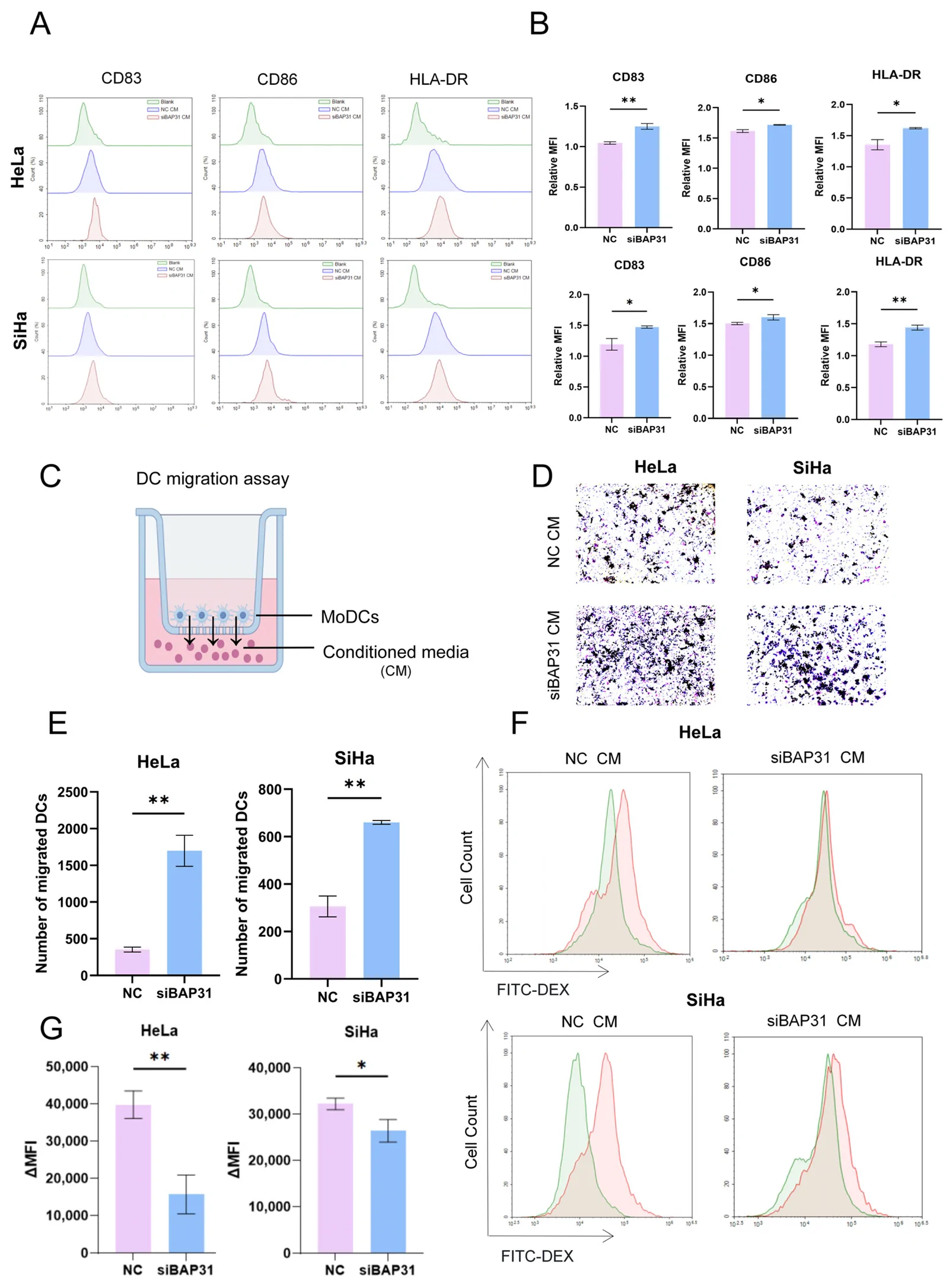
BAP31 silencing in CC cells modulates DC maturation and function. (**A**) Flow cytometry was used to detect the MFI of DC maturation markers CD83, CD86, and HLA-DR. (**B**) Quantitative analysis of the relative MFI of DC maturation markers. (**C**) Schematic illustration of the Transwell migration assay used to evaluate DC migration in response to tumor cell CM. (**D**) Representative images of migrated DCs after treatment with NC CM or siBAP31 CM in the Transwell migration assay. (**E**) Quantification of migrated DCs after treatment with different CM. (**F**) Flow cytometric histograms showing DC phagocytic activity assessed by FITC-dextran uptake. (**G**) Quantification of ΔMFI in the FITC-dextran uptake assay. * *p* < 0.05, ** *p* < 0.01.

**Figure 4:**
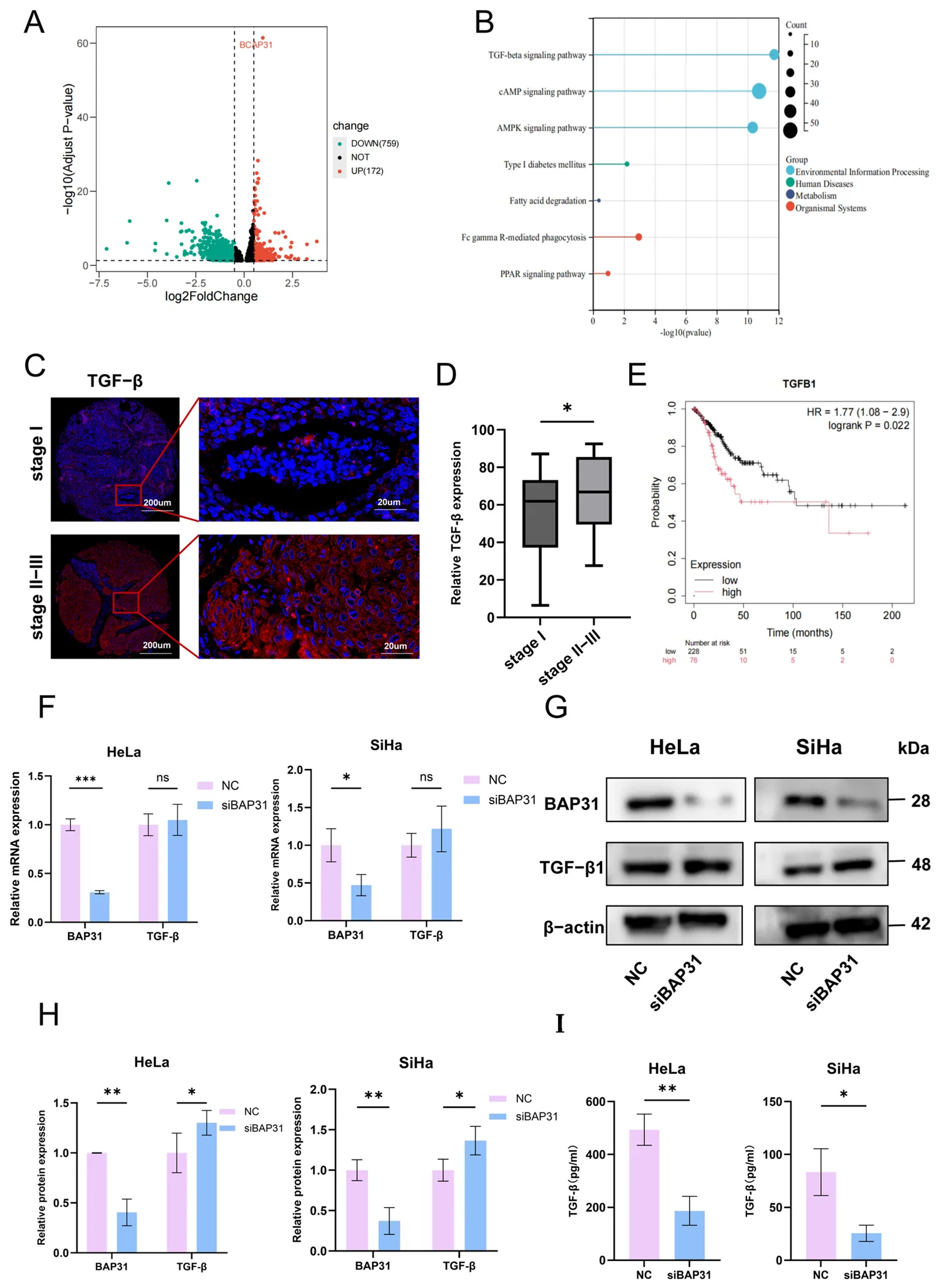
BAP31 is associated with TGF-β expression, prognosis, and protein secretion in CC. (**A**) Volcano plot showing differentially expressed genes (DEGs) between BAP31-high and -low CC tissues from the TCGA database. DEGs were defined as |log2FC| > 0.5 and FDR < 0.05. A total of 931 DEGs were identified, including 172 upregulated and 759 downregulated genes. The horizontal dashed line indicates FDR = 0.05, and the vertical dashed lines denote log2FC = −0.5 and 0.5, corresponding to the cutoff for gene expression fold change. (**B**) KEGG pathway enrichment analysis of the identified DEGs. Pathways with an adjusted *p* value < 0.05 were considered significantly enriched. (**C**) Representative mIF staining of TGF-β expression in CC tissues at different clinical stages (*n* = 86). (**D**) The expression of TGF-β in different stages of CC tissues (*n* = 86). (**E**) Kaplan–Meier overall survival analysis of CC patients according to TGF-β1 expression using the Kaplan–Meier Plotter (*n* = 304). Patients were stratified based on percentile ranking (HR = 1.77, 95% CI: 1.08–2.90; log-rank *p* = 0.022). (**F**) qRT-PCR analysis of TGF-β mRNA expression in BAP31-knockdown HeLa and SiHa cells. (**G**) WB analysis of precursor TGF-β1 protein after BAP31 knockdown. (**H**) Quantitative analysis of relative gray values of WB bands. (**I**) ELISA for the level of secreted TGF-β1 in the culture supernatants. * *p* < 0.05, ** *p* < 0.01, *** *p* < 0.001, ns, not significant.

**Figure 5:**
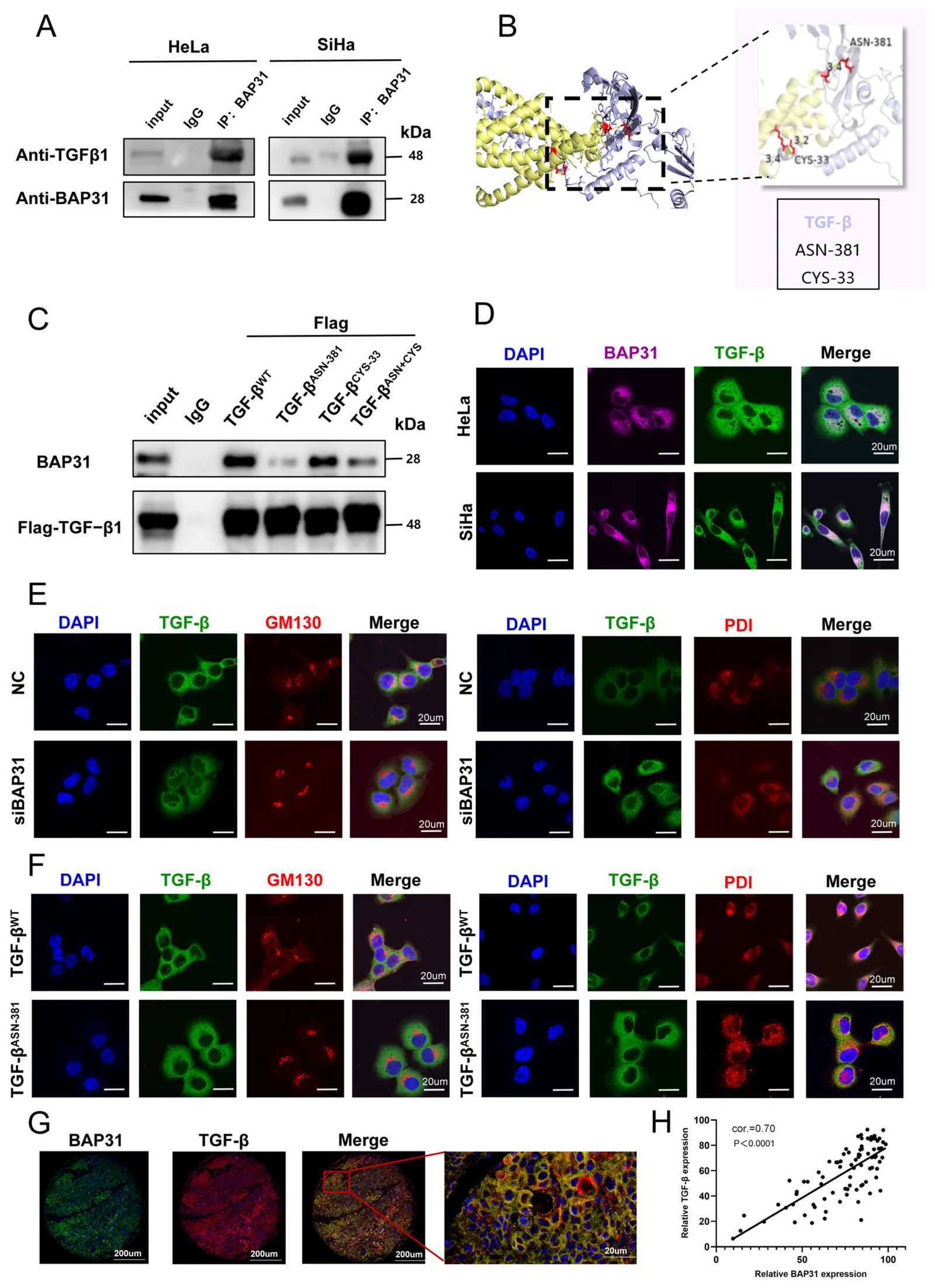
BAP31 regulates TGF-β trafficking and maturation through an Asn-381 interaction. (**A**) Co-IP assays showing the association between BAP31 and TGF-β. (**B**) Predicted interaction model of the BAP31–TGF-β complex based on AlphaFold multimer modeling. (**C**) Co-IP analysis of the association between BAP31 and TGF-β following point mutations at Asn-381 and Cys-33. (**D**) IF assay for the co-localization of BAP31 and TGF-β. (**E**) The co-localization of TGF-β with the ER and Golgi apparatus in control and BAP31-knockdown cells. (**F**) The co-localization of TGF-β mutants with the ER and Golgi apparatus. (**G**) Co-localization analysis of BAP31 and TGF-β based on tissue microarrays (*n* = 86). (**H**) Correlation analysis of BAP31 and TGF-β expression levels in CC tissues (*n* = 86). Correlation was assessed using Spearman’s rank correlation analysis.

**Figure 6:**
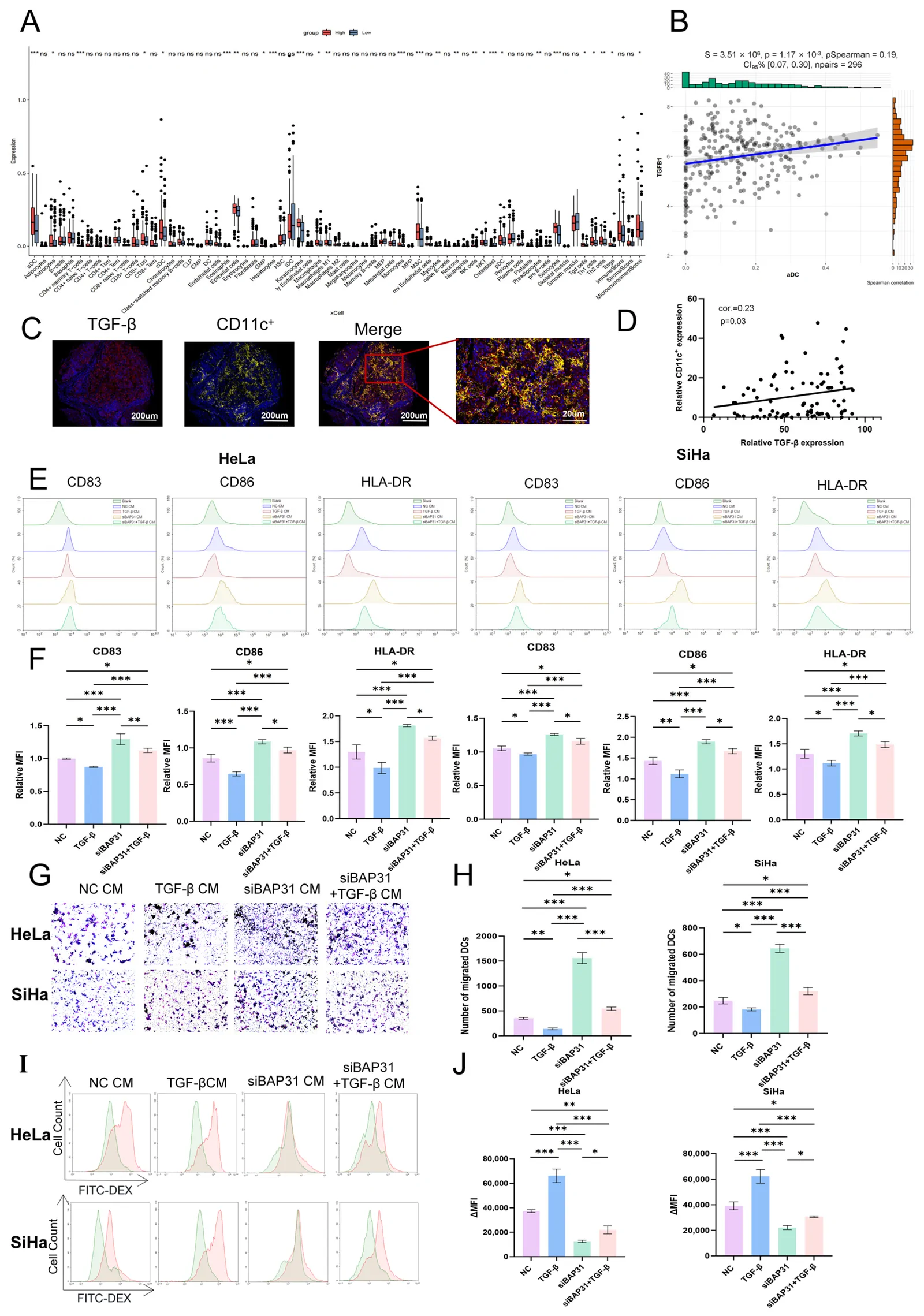
Functional contribution of TGF-β to dendritic cell regulation in CC. (**A**) Distribution of immune and stromal cells in CC tissues with high/low TGF-β expression analyzed by xCell algorithm (*n* = 296). (**B**) Correlation between TGF-β expression and DC infiltration. Correlation was assessed using Spearman’s rank correlation analysis. (**C**) mIF staining for TGF-β and CD11c^+^ (nuclei stained with DAPI). (**D**) Correlation between TGF-β expression and CD11c^+^ cell density in CC tissues (*n* = 86). Correlation was assessed using Spearman’s rank correlation analysis. (**E**) Flow cytometry detection of relative fluorescence intensity of DC maturation markers. (**F**) Quantification of the relative fluorescence intensity of CD83, CD86, and HLA-DR in DCs under the indicated treatment conditions. (**G**) Representative images and quantification of migrated DCs in the Transwell migration assay under the indicated treatment conditions. (**H**) Quantification of migrated DCs in the Transwell migration assay. (**I**) Representative flow cytometric histograms of FITC-dextran uptake by DCs under the indicated treatment conditions. (**J**) Quantification of DC phagocytic activity based on ΔMFI in the FITC-dextran uptake assay. * *p* < 0.05, ** *p* < 0.01, *** *p* < 0.001, ns, not significant.

**Table 1:** Relationship between BAP31 expression and clinicopathological parameters in CC.

Clinical Parameters	BAP31-High (*n* = 43, %)	BAP31-Low (*n* = 43, %)	Total (*n* = 86,%)	*p*-Value
Age				
<50	23 (26.74%)	25 (29.07%)	48 (55.81%)	0.83
≥50	20 (23.26%)	18 (20.93%)	38 (44.19%)	
Stage				
I	24 (27.91%)	37 (43.02%)	61 (70.93%)	0.0039
II-III	19 (22.09%)	6 (6.98%)	25 (29.07%)	
T classification				
T1	25 (29.07%)	37 (43.02%)	62 (72.09%)	0.0074
T2-T3	18 (20.93%)	6 (6.98%)	24 (27.91%)	

Caption: Data are presented as *n* (%). Associations between BAP31 expression and clinicopathological characteristics were analyzed using the χ^2^ test or Fisher’s exact test, as appropriate. A two-sided *p* < 0.05 was considered statistically significant.

## Data Availability

The data used to support the findings of this study are available from the corresponding authors upon request.

## Supplementary Materials

The following are available online at https://www.mdpi.com/article/10.3390/cells15191765/s1, Table S1: The siRNAs sequences, Table S2: Primer sequences, Table S3: Primary and secondary antibodies and dilution ratio, Table S4: Primary antibodies and dilution ratio, Table S5: List of antibodies and isotype controls used in this study, Figure S1: BAP31 knockdown impairs the ER-to-Golgi trafficking of TGF-β in cervical cancer cells, Figure S2: (A) Correlation between TGFB1 expression and immune cell infiltration in CESC. (B) Spearman correlation between TGFB1 expression and DC infiltration score in CESC.

## Abbreviations

The following abbreviations are used in this manuscript:

CC	Cervical cancer
BAP31	B-cell receptor-associated protein 31
ER	endoplasmic Reticulum
DC	Dendritic cell
TGF-β	Transforming growth factor-beta
TMA	Tissue microarray
mIF	Multiplex immunofluorescence
TCGA	The Cancer Genome Atlas
GTEx	Genotype-Tissue Expression
HRs	Hazard ratios
CIs	Confidence intervals
GEO	Gene Expression Omnibus
DEGs	Differentially expressed genes
KEGG	Kyoto Encyclopedia of Genes and Genomes
siRNAs	small interfering RNAs
CM	Conditioned medium
NC	Negative control
siBAP31	small interfering RNAs targeting BAP31
ELISA	Enzyme-linked immunosorbent assay
NCBI	National Center for Biotechnology Information
iDCs	immature dendritic cells
mDC	mature dendritic cells
qRT-PCR	Quantitative Real-Time Reverse Transcription PCR
