# Supplementary material for: BAP31 Facilitates TGF-β Trafficking and Secretion and Promotes Dendritic Cell Dysfunction in Cervical Cancer
Source: Cells. 2026 Sep 27;15(19):1765. doi: 10.3390/cells15191765 (PMC13663929; doi:10.3390/cells15191765)
Supplement: Supplementary file 1 [file cells-15-01765-s001.zip › Supplementary Material.pdf]

Supplementary Table S1. The siRNAs sequences

Name	sense (5' > 3')	antisense (5' > 3')
siBAP31	GGU GAA CCU CCA GAA CAA UTT	AUU GUU CUG GAG GUU CAC CTT
NC	UUCUCCGAACGUGUCACGU	ACGUGACACGUUCGGAGAA

Supplementary Table S2. Primer sequences

Gene	Forward primer (5′→3′)	Reverse primer(5′→3′)
BAP31	CGGCTGGTGGAGTTGTTAGT	CGGGATTGTTCTGGAGGT
TGF-β	GGCCAGATCCTGTCCAAGC	GTGGGTTTCCACCATTAGCAC
β-actin	CTCCCTCCATCCTGGCCTCGCTGT	CTTCTACCAAGACAAAAATGCTCA

Supplementary Table S3. Primary and secondary antibodies and dilution ratio

Target	Company	Cat.No.	Dilution ratio
BAP31	Abcam, UK	ab109304	1：5000
TGF-β	Selleck, USA	F1624	1：1000
β-actin	Proteintech	60008-1-Ig	1：5000
anti-mouse HRP secondary antibody	Immunoway	RS0001	1：3000
anti-rabbit HRP secondary antibody	Immunoway	RS0002	1：3000
His tag Recombinant Rabbit mAb	Bioss,China	bsm-33004R	1：8000
Flag Tag Recombinant Rabbit mAb	Bioss,China	bsm-33346R	1：8000

Supplementary Table S4. Primary antibodies and dilution ratio

Target	Company	Cat.No.	Dilution ratio
BAP31	Abcam, UK	ab109304	1：100
TGF-β	Origene, USA	TA506583	1：100
PDI	CST, USA	#3501	1：100
GM130	CST, USA	#12480	1：3200

Supplementary Table S5. List of antibodies and isotype controls used in this study

Antibodies	Source	Catalog No.
PE-conjugated anti-CD83	Elabscience	#E-AB-F0993D
APC-conjugated anti-HLA-DR	Elabscience	#E-AB-F1111E
PE/Cyanine7-conjugated anti-CD86	Elabscience	#E-AB-F1012H
FITC-conjugated anti-CD11b	Elabscience	#17-1419-42
PE-conjugated anti-CD141	Selleck	#H1967
PE Mouse IgG1, κIsotype Control [MOPC-21]	Elabscience	#E-AB-F09792D
APC Mouse IgG2a, κIsotype Control [C1.18.4]	Elabscience	#E-AB-F09802E
PE/Cyanine7 Mouse IgG1, κIsotype Control[MOPC-21]	Elabscience	#E-AB-F09792H
FITC Rat IgG2b, κ Isotype Control[LTF-2]	Elabscience	#E-AB-F09842C
PE Mouse IgG1, κ Isotype Control[MOPC-21]	Selleck	#G4187

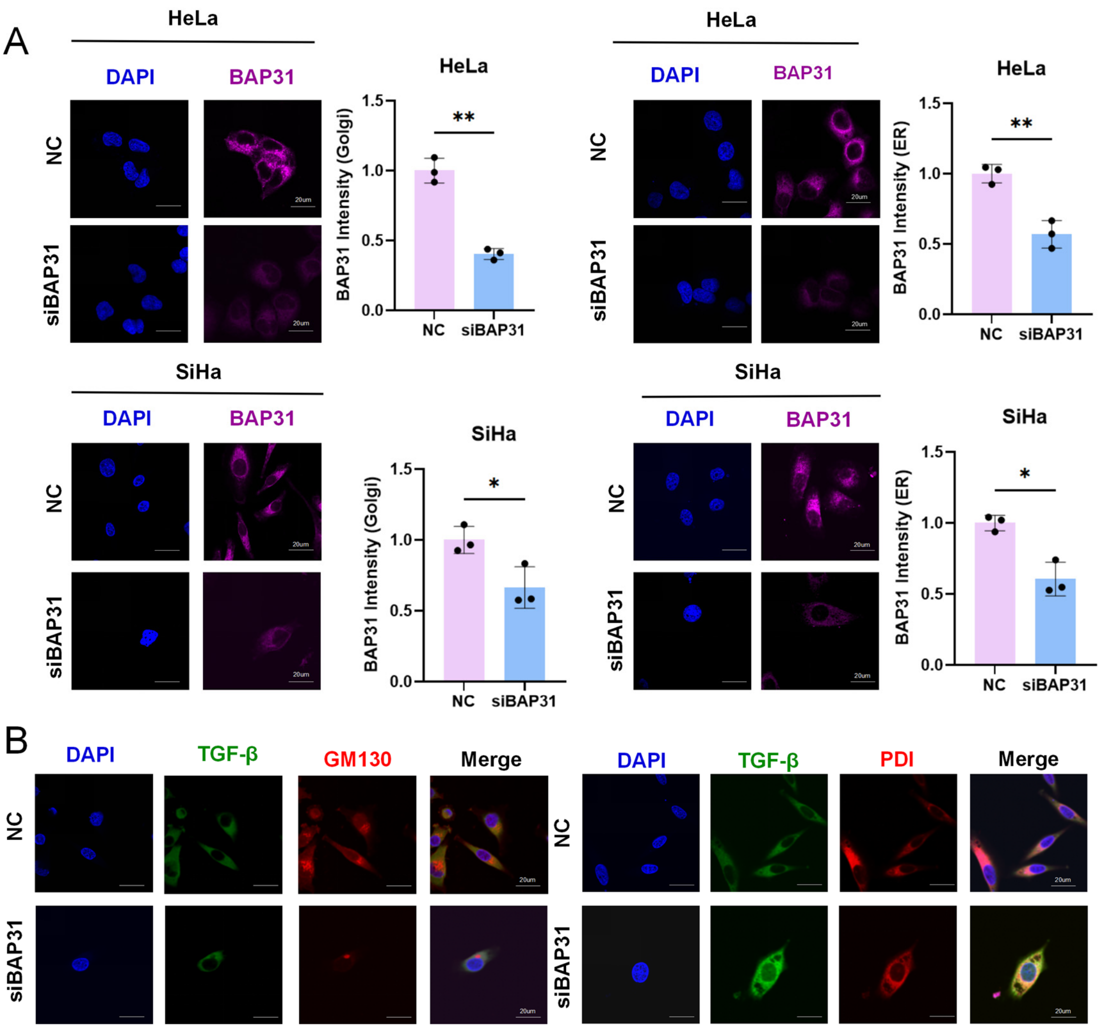

Supplementary Figure S1. BAP31 knockdown impairs the ER-to-Golgi trafficking of TGF-β in cervical cancer cells. (A) Representative immunofluorescence images and corresponding quantification of BAP31 expression in HeLa and SiHa cells transfected with control NC or siBAP31. (B) Co-localization of TGF-β with the ER marker PDI and the Golgi marker GM130 in SiHa cells after BAP31 knockdown.

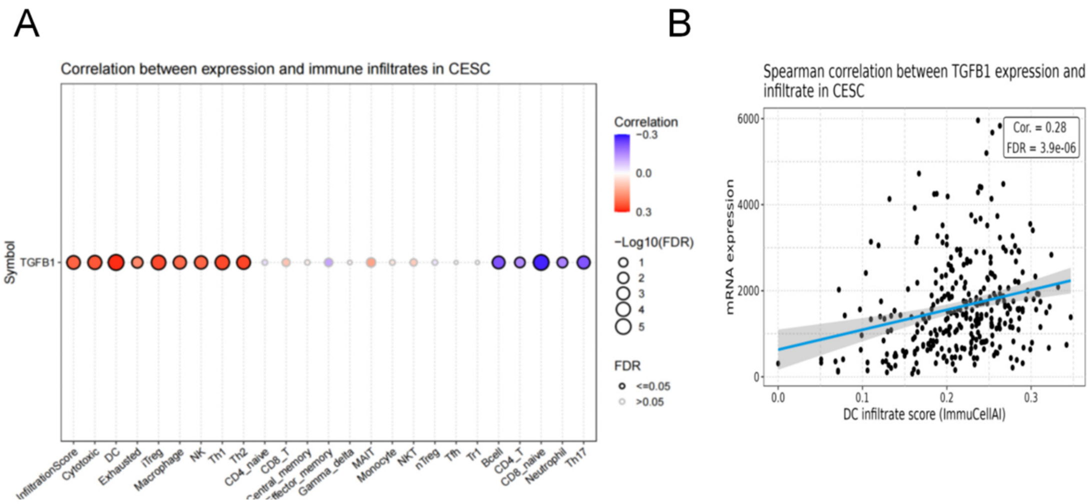

Supplementary Figure S2. (A) Correlation between TGFB1 expression and immune cell infiltration in CESC. (B) Spearman correlation between TGFB1 expression and DC infiltration score in CESC.
